# Storage Drives Alterations of Proteomic and Protein Structural Properties in Rice (*Oryza sativa* L.)

**DOI:** 10.3390/foods11213541

**Published:** 2022-11-07

**Authors:** Qian Wang, Dong Zhang, Jianlei Liu, Bo Shang, Xiaoliang Duan, Hui Sun

**Affiliations:** Academy of National Food and Strategic Reserves Administration, Beijing 100037, China

**Keywords:** rice, storage, proteomics, TMT-labeled, structural

## Abstract

Rice quality changes during storage. However, few studies have reported the difference in protein structure between the *indica* and *japonica* varieties of rice during storage. The current research characterized the structural properties of the rice protein, and further investigated the proteomic profiles of Jianzhen 2 (*indica* rice) and Nanjing 9108 (*japonica* rice) during storage using the TMT labeling method. A significant reduction in free sulfhydryl content and an increase in disulfide bonds content and surface hydrophobicity were observed in both varieties after storage. The results of FTIR indicated that the changes in the protein’s secondary structure of Nanjing 9108 (*japonica* rice) were more significant than in Jianzhen 2 (*indica* rice). A total of 4039 proteins in Nanjing 9108 and 4301 proteins in Jianzhen 2 were identified by TMT-labeled proteomics analysis in this study. Significantly, changes were detected in 831 proteins in Nanjing 9108, while only in 60 proteins in Jianzhen 2. Protein processing in endoplasmic reticulum, starch, and sucrose metabolism were both accelerated in both varieties, while oxidative phosphorylation in mitochondria, glycolysis, fatty acid metabolism, and glutathione metabolism were enhanced in Nanjing 9108 (*japonica* rice). This study provides insight into the proteomic changes and protein structure in rice induced by storage.

## 1. Introduction

As an important raw material for the food industry and a staple food for 50% of the world’s population, rice (*Oryza sativa* L.) is a vital crop worldwide [1]. It is extensively cultivated in China, where it is significantly consumed. According to the latest report released by United Nations’ Food and Agricultural Organization (FAO), the global rice production in 2020/21 was estimated at 501.1 million tons, with China accounting for approximately 40%. It is stored for a short or long time after harvest in order to maintain a continuous supply to the consumers. However, storage usually leads to deterioration in eating quality, which is often caused by changes in physical and chemical composition [1,2,3].

Protein, as the second largest component of rice grain (approximately 4~14%), is inseparable from the hardness and viscosity of rice [4,5,6]. It is also influenced by temperature or humidity and undergoes chemical and physical changes after storage [7,8]. The content and conversion of free sulfhydryl and disulfide bonds are the critical factors influencing the protein quality of aged rice. Free sulfhydryl content is generally decreased in stored rice, while the disulfide bond content is increased [7,8,9]. Guo et al. [10] concluded that sulfhydryl groups that were oxidized to disulfide bonds in globulin lead to a decrease in α-helix content of stored rice protein. Therefore, whether the protein structure of different rice varieties exhibits consistent changes during storage is unknown.

Proteomics is one of the primary fields in the “omics” sciences, which include gel-based and mass spectrometry (MS)-based approaches to characterize proteins on a large scale in a single cell, tissue, or whole organism [11]. With the development of new MS techniques, a gel-free, MS-based quantitative proteomic approach has already become the mainstream method to analyze protein profiles in biological samples. Label-free and isotope labeling approaches are the most popular methods in MS-based quantitative proteomics [11]. The isotope labeling approaches mainly include TMT labeling developed by Thermo Fisher Scientific (CA, USA) and iTRAQ labeling by Applied Biosystems (CA, USA). Proteomics analysis has been successfully utilized for the characterization of proteins in rice. For instance, Liu et al. [12] revealed the change in protein profile during rice yellowing using TMT labeling proteomics, demonstrating that the heat shock protein was significantly up-regulated. Using a quantitative label-free proteomic approach, Xiao et al. [13] differentiated conventional and organic rice. Zhao et al. [7] performed the proteomics analysis (non-labeled) and investigated the structural properties of Daohuaxiang stored for 300 days under different temperatures, and demonstrated that a higher temperature aggravated the changes in protein structure in rice. However, the comparative protein profiles of different rice varieties during storage have not been reported.

In the current study, TMT-labeled proteomics was utilized to explore the alters in protein profiles of freshly harvested and stored Jianzhen 2 (*indica* rice) and Nanjing 9108 (*japonica* rice), which are rice varieties with large planting areas in southern China due to their excellent eating quality. The structural properties of fresh and stored rice protein were further investigated based on free sulfhydryl content, number of disulfide bonds, surface hydrophobicity (H_0_), and Fourier transform infrared spectroscopy (FTIR). This analysis fills the research gap in rice omics (metabolomics and lipidomics) [14,15,16] and provides a comprehensive insight into the role of proteins in rice quality deterioration.

## 2. Materials and Methods

### 2.1. Regents, Material, and Storage Condition

TMT^®^ Mass Tagging Kits and Reagents, Ellman’s reagent, acetonitrile, methanol, acetone, formic acid, and ultrapure water were purchased from Thermo Fisher Co. Ltd. (Shanghai, China). Ammonium bicarbonate (purity ≥ 99%), dithiothreitol (DTT, purity ≥ 98%), ammonium hydroxide, triethylammonium bicarbonate (TEAB, pH 8.5 ± 0.1, 1.0 M), trifluoroacetic acid (TFA, purity ≥ 99%), iodoacetamide (IAM, Purity ≥ 99%), and ANS (1-anilinonaphthalene-8-sulfonic acid) were obtained from Sigma-Aldrich (Shanghai, China). Protein quantification kit (Bradford assay) was obtained from Beyotime Institute of Biotechnology (Beijing, China). Trypsin Gold (V5280, MS grade) and ProteoMiner Protein Enrichment Small-Capacity Kits were collected from Promega (Beijing, China) and Bio-Rad (Shanghai, China), respectively.

Typical *indica* rice (Jianzhen 2) and *japonica* rice (Nanjing 9108) were harvested in Hubei and Jiangsu provinces of southern China in October 2018. After the moisture content in the samples was reduced to about 13%, they were packaged and kept in an artificial weather box (1 m^3^, 20 °C, relative humidity of 27%) for 540 days until March 2020. Freshly harvested samples were stored at −80 °C until analysis. Jianzhen 2 and Nanjing 9108 are abbreviated as JZ and NJ, respectively. The stored and fresh groups were designated as S and F, respectively. Comparison between JZS vs. JZF and NJS vs. NJF was conducted via subsequent experiments. The eating quality of stored samples was 74.7 (JZS) and 65.3 (NJS), while that of fresh samples was 82.1 (JZF) and 81.0 (NJF).

### 2.2. Structural Characteristics

#### 2.2.1. Extraction of Rice Protein

The rice sample degreased with n-hexane (*w*/*v* = 1:5) was added to 1.0 M NaOH solution (*w*/*v* = 1:9), oscillated for 4 h (25 °C), and centrifuged to obtain the supernatant (6000× *g*, 30 min). The supernatant was regulated to pH 5.5 with 2 M HCl to induce protein precipitation. The protein precipitate was freeze-dried after washing with deionized water three times.

#### 2.2.2. Surface Hydrophobicity (H_0_)

The H_0_ of rice protein was measured with ANS according to the modification of Wu et al. [17]. The freeze-dried rice protein was dissolved in a phosphate buffer (pH 7.0, 0.01 M). ANS solution (20 μL of 8 mmol/L) was added to 2 mL of adjusted protein solution (0.02~0.1 mg/mL) and stored in the dark for 10 min after mixing with a Vortex-Genie 2T oscillator (Scientific Industries, New York, NY, USA). The fluorescence intensity was measured with a multimode plate reader (EnSpire, PerkinElmer, Waltham, MA, USA) at an excitation wavelength of 390 nm (emission wavelength: 470 nm). The slope of the linear equation fitted by fluorescence intensity to protein concentration was defined as H_0_.

#### 2.2.3. Free Sulfhydryl and Disulfide Bond

The content of free sulfhydryl and disulfide bond were determined based on the molar absorptivity of Ellman’s reagent (ε = 13,600 L/(mol⋅cm)). By substracting the free sulfhydryl content from the total sulfhydryl content (reduction with β-mercaptoethanol), the disulfide bonds were calculated as follows:

Disulfide bond content = (total sulfhydryl content − free sulfhydryl content)/2(1)

#### 2.2.4. FTIR

A Fourier transform micro-infrared spectrometer (Nicolet iN10, Thermo Scientific, MA, USA) was used to obtain the spectrum (32 scans, 4000–400 cm^−1^) according to the modified method of Zhao et al. [7]. The infrared spectra of amide I bands (1600~1700 cm^−1^) of protein were analyzed with PeakFit 4.12 software (Sea-Solve Software Inc., New York, NY, USA). The percentages of different secondary structures were calculated according to the peak areas of each sub peak after curve smoothing, baseline correction, Gaussian deconvolution, and second derivative fitting.

### 2.3. Structural Characteristics

#### 2.3.1. Isolation of and Protein Extraction

Protein extraction was performed as previously described [18,19]. Rice samples (0.5 g) were ground into powder in liquid nitrogen and then transferred into a centrifugal tube. The sample was mixed with 4 mL of lysis buffer (with 10 mM TEAB, 100 mM DTT, and 4% SDS) and sonicated for 5 min. After reacting at 95 °C for 8 min, the lysate was centrifuged at 4 °C (12,000× *g*, 15 min). The upper layer was collected and reduced with 10 mM DTT (56 °C, 1 h), followed by the addition of 1 mL of IAM to alkylate proteins. The mixture was kept in the dark for 1 h (25 °C). Subsequently, 20 mL of pre-cooled acetone was added to the mixtures, vortexed vigorously, and incubated at −20 °C (2 h). Samples were then centrifuged at 4 °C (12,000× *g*, 15 min), and the precipitation collected. After washing with 1mL of precooled acetone 3 times, the residue was redissolved with 100 μL of dissolution buffer (8 M urea and 100 mM TEAB).

#### 2.3.2. Digestion and TMT Labeling

Protein digestion and TMT labeling were performed as described by Kachuk, Stephen and Doucette [18], and Wiśniewski et al. [19]. For digestion, trypsin was added (trypsin/protein = 1:50) for the first digestion (37 °C, 4 h). The second digestion was performed overnight with trypsin (1:100 trypsin/protein mass ratio) and calcium chloride solution (0.02%) at 4 °C. Formic acid was added to the digested protein to adjust the pH (2−3). After centrifugation (12,000× *g*, 5 min), the supernatant was desalted using an active Sep Pak C18 desalting column (Waters, MA, USA). The eluents were collected and lyophilized for 24 h. Then, 100 μL of TEAB buffer (0.1 M) was pipetted to redissolve the peptide residues, followed by the addition of 41 μL of TMT labeling reagent (acetonitrile-dissolved). Next, the mixture was oscillated for 2 h at room temperature, and the reaction was stopped by 8% ammonia. Finally, the labeled peptides from all samples were desalted and lyophilized after being mixed with equal volumes.

#### 2.3.3. Separation of Peptide Fractions

The procedure of peptide fractionation was performed with a Rigol L3000 HPLC system (RIGOL Technologies, Beijing, China) using a BEH-C18 (5 µm, 4.6 × 250 mm) column. Mobile phase (A: 2% acetonitrile, adjusted pH to 10.0 with ammonium hydroxide; B: 98% acetonitrile) at the flow rate of 1 mL/min was applied to fractionate the peptides. The lyophilized peptides were dissolved with mobile phase A and centrifuged (12,000× *g*, 10 min), followed by HPLC of 1 mL of supernatant. The gradient elution was carried out as seen in Table S1. The column temperature was set to 45 °C. All fractions were vacuum freeze-dried for further analysis.

#### 2.3.4. LC-MS/MS Analysis

Proteomics analysis was conducted using an EASY-nLC^TM^ 1200 UHPLC system (Thermo Fisher Scientific, Waltham, MA, USA) combined with a Q-Exactive^TM^ HF-X mass spectrometer (Thermo Fisher Scientific, Waltham, MA, USA). Lyophilized peptides were redissolved with 0.1% formic acid, followed by injection of 1 μg of solution which was injected into a self-made analytical column (15 cm × 150 μm, 1.9 μm). Mobile phases A (2% acetonitrile, 0.1% formic acid) and B (98% acetonitrile, 0.1% formic acid) were used to develop a gradient elution (Table S2). The fractionated peptides were analyzed with a Q-Exactive^TM^ HF-X mass spectrometer (Thermo Fisher Scientific, Waltham, MA, USA) with a Nanospray Flex^TM^ ion source. The spray voltage and transport capillary temperature were set at 2.1 kV and 320 °C, respectively. The m/z scan range was of 350–1500 for the full scan mode. The intact peptides were detected via Orbitrap MS at a resolution of 60,000. Peptides were then selected for MS/MS mode using an NCE (28). The fragments were detected at a resolution of 30,000. The data-dependent acquisition mode was used after 1 full scan, followed by 20 MS/MS scans. The dynamic exclusion was 20 s. The automatic gain control target value was set at 3 × 10^6^, and a maximum ion injection time was set at 20 ms.

#### 2.3.5. Identification and Quantitation of Protein

The Proteome Discoverer 2.2 (Thermo Fisher Scientific, Waltham, MA, USA) software and oryza_sativa_l_uniprot_2020_7_2.fasta (96036 sequences) database was used to acquire the results of peptides MS/MS. The search parameters are shown in Table S3. In order to improve accuracy, the results were further filtered using Proteome Discoverer software. At least 1 unique peptide was contained in each identified protein. The identified peptide spectrum matches and protein were retained and analyzed with a false discovery rate (FDR) no greater than 1.0%.

#### 2.3.6. Differentially Expressed Proteins and Functional Analysis of Proteins

The proteins with |log_2_FC| > 0.26 (FC > 1.2 or FC < 0.83) and *p* < 0.05 between freshly harvested and stored rice were considered as differentially expressed proteins (DEPs). DEPs were used for cluster heat map visualization and Kyoto Encyclopedia of Genes and Genomes (KEGGs) enrichment analysis [19]. Gene Ontology (GO) and InterPro (IPR) functional analysis were carried out using the InterProScan program against the non-redundant protein database [18]. The databases of Clusters of Orthologous Groups (COGs) and KEGGs were used to analyze the protein family and pathway, respectively.

### 2.4. Statistical Analysis

The Metware database (Metware, Hubei, China) was adopted to analyze the data and identify the proteins. The protein quantitation results were statistically analyzed by *t*-test. The Metware platform (https://cloud.metware.cn/, accessed on 28 May 2022) was used to perform the partial least squares-discriminant analysis (PLS-DA).

## 3. Results and Discussion

### 3.1. Structural Properties

#### 3.1.1. Sulfhydryl and Disulfide Bonds Content

**Figure 1:**
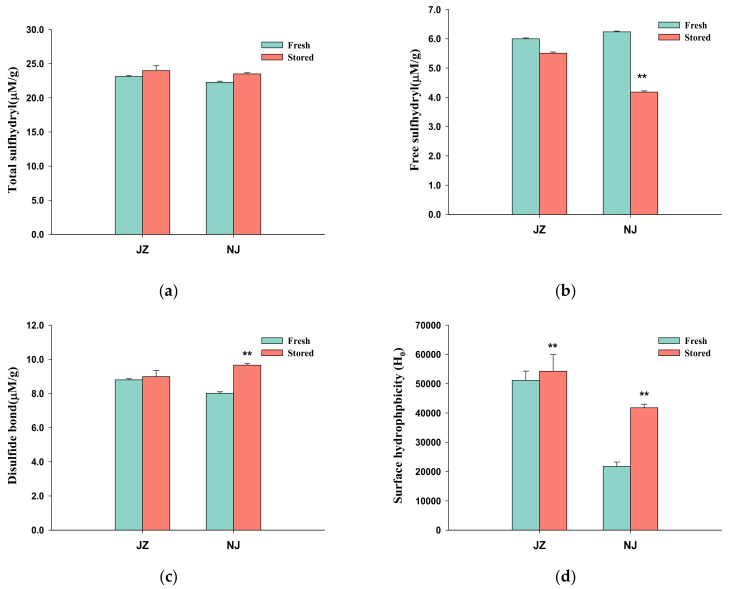
Effect of storage on the protein structural properties in rice. (**a**) Free sulfhydryl; (**b**) Total sulfhydryl; (**c**) Disulfide bonds; (**d**) Surface hydrophobicity H_0_. (*n* = 3, **: α = 0.01).

Sulfhydryl is the most active amino acid side-chain group of protein degradation [20]. The protein oxidation level is always evaluated by the abundance of free sulfhydryl and disulfide bonds [17,21,22]. The free sulfhydryl content decreased significantly, and the disulfide bond content increased significantly in the stored samples (Figure 1). The content of free sulfhydryl in three Iranian rice varieties decreased by more than 10% at 37 °C after 6 months [23]. The oxidation of the sulfhydryl group can be divided into reversible and irreversible types. Disulfide bonds were formed by reversible oxidation of sulfhydryl groups, while non-disulfide covalent bonds, such as sulfinic acid, were generated via irreversible oxidation. This reversible oxidation between free sulfhydryl and disulfide bonds generally contributes to the loose spatial structure of protein and the reduction in rice viscosity [24].

#### 3.1.2. Surface Hydrophobicity H_0_

The surface hydrophobicity of proteins can affect the protein conformational structural changes. ANS is a special fluorescent probe that can bind with surface hydrophobic groups on protein to emit fluorescence, and the fluorescence intensity is related to the number of binding sites. The surface hydrophobicity (H_0_) values of protein in JZF and NJF were 51,108 and 21,756, respectively, and the H_0_ of the stored rice protein was higher than that of the fresh one in both varieties (Figure 1d). Protein conformational changes, such as stretching and unfolding, were associated with lipid oxidation during storage. The changes expose the aromatic amino acid side-chain groups and hydrophobic aliphatic buried inside, enhancing the surface hydrophobicity of the protein [7,25]. On the other hand, the increase in surface hydrophobicity may also be due to the dissociation of protein subunits during storage [26]. The change in H_0_ of JZ was less than that of NJ, which indicates a difference in the protein structure of the two varieties, suggesting that *indica* rice is more tolerant to storage than *japonica* rice.

#### 3.1.3. FTIR

Amide I bands (1700–1600 cm^−1^) determined by FTIR can be used to quantitatively analyze the secondary structure of proteins. Therefore, it is widely used in the study of rice protein and polypeptide structure [7,17,27]. The impact of storage on the secondary structure of rice protein in two varieties is shown in Table 1. The original FT-IR spectra of the protein samples are displayed in Figure S1. α-Helix and β-sheet belong to periodic structure, while β-turn and random coil do not. The former two reflect the order of protein molecules, while the latter two reflect the loosening of protein molecules. Storage disrupts the secondary structure. NJ (*japonica* rice) exhibited a more severe transformation than JZ (*indica* rice). A slight rise in β-sheet and a decrease in α-helix and β-turn were observed in JZ (*indica* rice). In contrast, the NJ (*japonica* rice) showed the opposite changes of ordered and disordered structure, indicating that mutual transformation [28]. We speculated that some of the α-helixes and β-sheets turn into disordered random coils as the reduction in β-sheet was greater than the increase in relative content of β-turn. Singh and Sogi [29] reported that the β-sheet was more easily affected by the environment and processing conditions than other secondary structures. Consistently, the reduction in α-helix was lower than that in β-sheet in the protein secondary structural change in NJ. The raise of the loose structure and the loss of the ordered structure during rice storage were attributed to protein oxidation induced by α-helix [21]. Finally, the increase in protein disorder exposes the hydrophobic residues inside to the surface of the protein, which further leads to the poor eating quality of the rice [7].

### 3.2. TMT-Labeled Proteomics

#### 3.2.1. Protein Identification in Both Rice Varieties

**Figure 2:**
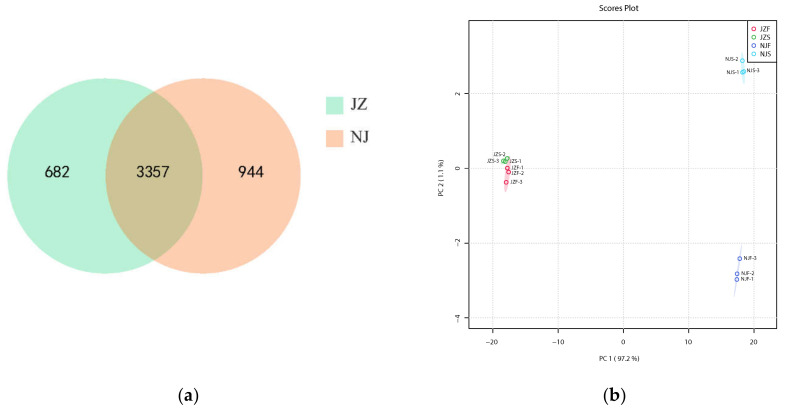
The protein variation between different varieties. (**a**) Venn plot; (**b**) PCA of protein profiles.

The LC-MS chromatograms are presented in Figure S2. The parent ion errors of rice peptide segments identified by mass spectrometry were less than 1.2%, as shown in Figure S3. The narrow range of mass tolerance distribution shows that the qualitative and quantitative results of protein are highly accurate, indicating the accuracy of MS. Figure S4 shows the length distribution of the identified protein segments. About 80% of the peptide segments shown in the figure are 7–20 amino acids, which are consistent with typical peptides, indicating that the sample preparation meets the requirements. A total of 22,318 peptides and 4039 proteins were identified in JZ, while 23,936 peptides and 4301 proteins were identified in NJ. The primary proteins in both varieties accounted for over 65% of the total proteins identified, with a mass range of 10–60 kDa (Figure S5). Further quantitative analysis revealed 4040 and 4301 quantifiable proteins in JZ and NJ, respectively. Among these proteins, 3357 were shared by the two varieties, and the specific proteins of JZ and NJ were 944 and 682, respectively (Figure 2a). The detailed information of the quantitative proteins is listed in Table S4.

PCA analysis was adopted to assess the variants in the protein profiles. Based on the PCA results, the four rice samples were clearly divided into four groups with PC1 (97.2%) and PC2 (1.1%). In particular, the separation was more visible in the NJ group than in the JZ group (Figure 2b).

#### 3.2.2. Identification of DEPs in Both Rice Varieties

**Figure 3:**
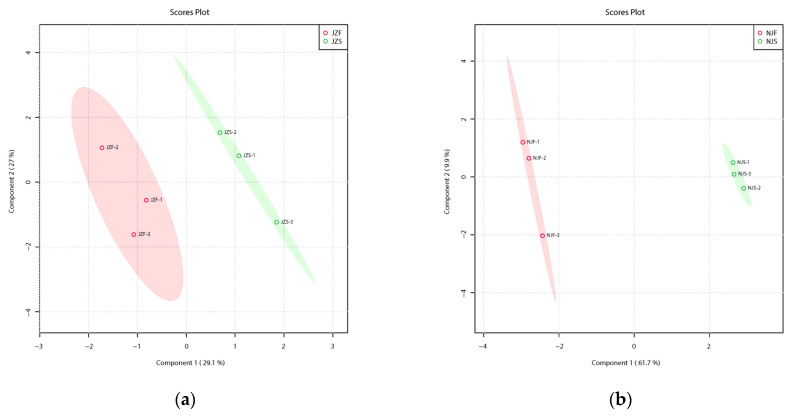

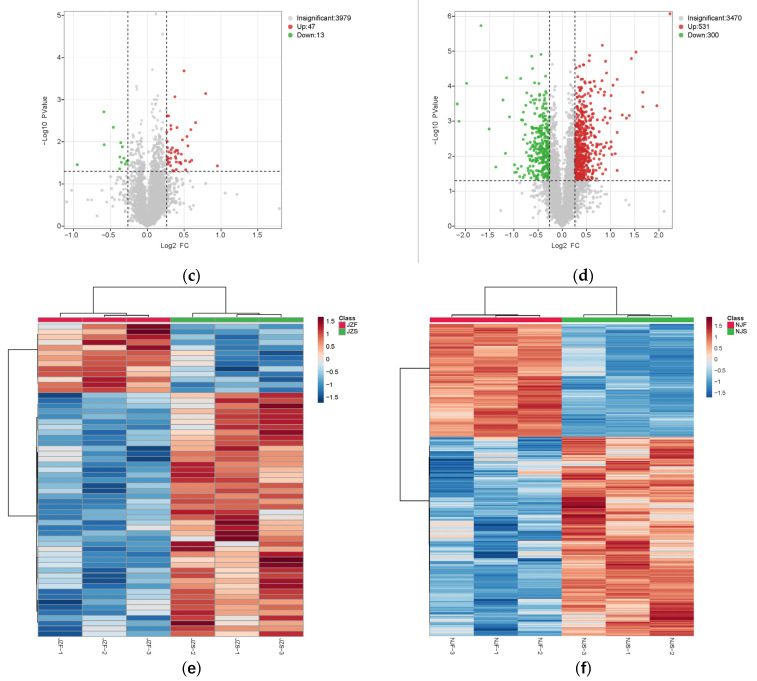
Protein variation across stored rice compared with fresh rice. (**a**) PLS-DA score plot of JZ. (**b**) PLS-DA score plot of NJ. (**c**) Volcano plot of DEPs in JZ. (**d**) Volcano plot of DEPs in NJ. (**e**) Heatmap for DEPs in JZ. (**f**) Heatmap for DEPs in NJ.

The individual proteins obtained at different storage times were contrasted via PLS-DA to identify DEPs in each comparison group individually. Figure 3a,b indicates a good separation between the groups before and after storage of the two rice varieties, suggesting that storage drives alterations of protein profile in rice.

NJ and JZ exhibited different protein expression patterns in freshly harvested and stored rice. Among 60 DEPs in NJ, 47 were up-expressed and 13 were down-expressed proteins in JZS compared with those in JZF (Figure 3c, Table S5a). Among the 831 DEPs identified in NJ, 531 were up-expressed and 300 were down-expressed in NJS compared with NJF (Figure 3d, Table S5b). These DEPs could differentiate freshly harvested and stored samples in both varieties (Figure 3e,f).

Table 2 lists all the overlapped DEPs in two varieties. Among these 15 DEPs, 12 proteins (Table 2, No.1-12) in two varieties changed similarly during storage, while the 3 others did not (Table 2, No.13-15). Intriguingly, the variation of those 12 proteins in NJ was more significant than in JZ. As shown in Figure S6a,b, among the top 20 DEPs, B9EZW1 and Q0IUY1 were the most significantly down-regulated and up-regulated proteins in JZ, with an FC fold of 0.517 and 1.931. A3BWT1 and A3CC78 were the most significantly down-regulated and up-regulated proteins for NJ, with an FC fold of 0.222 and 4.680.

#### 3.2.3. GO Analysis

**Figure 4:**
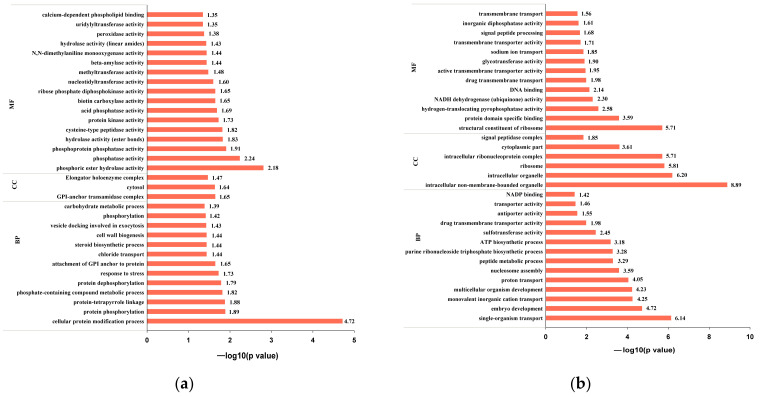
GO analysis of DEPs in JZ (**a**) and NJ (**b**).

Biological process (BP), cellular component (CC), and molecular function (MF) are three categories of gene ontology (GO). In the current study, 42 and 546 DEPs in JZ and NJ were annotated to 77 and 364 GO terms. Figure 4 displays the 14 and 37 significantly involved terms of JZ and NJ, respectively (*p* < 0.05).

The majority of the DEPs predominantly expressed in the JZ group were categorized under the BP terms in response to stress (four DEPs) and in response to chemical (two DEPs). The enriched BP terms were mainly related to nucleotidyltransferase activity (two DEPs) and sulfotransferase activity (one DEPs). The CC terms were related to the endoplasmic reticulum and its membrane (four DEPs).

In the NJ group, 28 terms were significantly related to BP. The most abundant were macromolecule modification (50 DEPs), cellular protein modification process (49 DEPs), cellular component organization or biogenesis (27 DEPs), single-organism transport (25 DEPs), cellular component biogenesis (17 DEPs), and macromolecular complex subunit organization (14 DEPs). The majority of the DEPs in the CC were classified into nucleosome (four DEPs) and mitochondrial matrix (three DEPs). The top four remarkable enriched MF terms were cysteine-type peptidase activity (10 DEPs), serine-type endopeptidase inhibitor activity (seven DEPs), and peptidyl-prolyl cis-trans isomerase activity (six DEPs).

#### 3.2.4. Comparative KEGG Pathway Analysis

In our study, 60 DEPs from 24 pathways were identified in JZ (*indica* rice), and 831 proteins from 45 pathways were identified in NJ (*japonica* rice) (Table S5). For JZ, the DEPs were mainly involved in galactose metabolism, purine metabolism, glycosylphosphatidylinositol (GPI)-anchor biosynthesis, ubiquitin-mediated proteolysis, protein processing in endoplasmic reticulum, and glycosphingolipid biosynthesis-globo and isoglobo series. For NJ, the DEPs were mainly involved in protein translation, oxidative phosphorylation, glycolysis, fatty acid metabolism, amino acid metabolism, glutathione metabolism, ribosome biogenesis in eukaryotes, plant hormone signal transduction, and maintenance of redox homeostasis.

The TMT-labeled proteomics analysis revealed that storage deteriorated the protein’s quality. Heat shock protein (HSP) is a series of molecular chaperones that can promote the folding of polypeptide chains into proteins with natural spatial conformation. It is a stress-responsive protein. Heat, drying, and oxygen stress always induce HSP generation to repair denatured proteins and prevent protein aggregation [7,30]. Putative HSP (70 kDa), class I HSP 2 (16.9 kDa), class II HSP (18.0 kDa), and class III HSP (18.6 kDa) were remarkably up-regulated in NJ, but not in JZ (Table 3, No.1-4). The HSP activity was increased due to the production of transferred protein precursors, variant proteins, and unstable proteins [31].

Protein disulfide isomerases (PDIs) are the principal catalysts in disulfide bond formation. PDIs not only catalyze the rearrangement of intra- and inter-chain disulfide bonds in proteins, but are also an essential part in the separation of glutenin and gliadin polypeptides in the endosperm [32,33]. The rice genomes generally encoded at least seven PDIs, including PDIL1-1/1-2/1-3/-1-4 and PDIL2-1/2-2/2-3 [34]. In this study, we found that PDIL1-2 was up-regulated in NJS and PDI (unknown) was down-regulated in JZS (Table 3, No.5-6). These results indicate that up-regulated PDIs may improve the tolerance to adverse environments and stability of the protein. Xu et al. [35] analyzed the sulfhydryl content and proteomics of aging coix seeds and reported an up-regulation in the levels of proteins related to resistance and antioxidant activity during storage. Hu [31] reported that HSP and PDIs catalyzed the formation of disulfide bonds between protein molecules in stored rice, which hindered the gelatinization of starch granules, further resulting in the deterioration of eating quality.

Glutathione S-transferase plays a crucial role in intracellular redox homeostasis and response to oxidative stress. It generally catalyzes the conjugation of glutathione and functions as a carrier of secondary metabolites to appropriate cellular localization [36,37]. This binding increases the hydrophobicity of the protein, which can be easily transported across the cell membrane and released externally. Thus, the level of glutathione S-transferase reflects the degree of antioxidant ability [12]. Glutathione S-transferase GSTU6 was significantly up-regulated in NJ (Table 3, No.7). Liu et al. [38] investigated the potential mechanisms of beef quality reduction under cold storage and reported that the decreasing water-holding capacity and quality was due to the inhibition of the glutathione metabolic pathway. Liu et al. [12] found that the changes of glutathione metabolism in rice were closely related to the yellowing of the stored samples. Our previous metabolomics study also found that the glutathione metabolic pathway was enhanced in rice after storage [14].

Oxidative phosphorylation is the process of ATP synthesis driven by the energy released by the oxidative decomposition of organic compounds. It transfers electrons and H^+^, and generates H_2_O and ATP in the presence of oxygen [39]. The down-regulation of oxidative phosphorylation in NJ (*japonica* rice) was attributed to the decrease in ATPase (No.8-12), NADH-ubiquinone oxidoreductase (No.13-14), and NADH dehydrogenase (ubiquinone) (No.15-21), which indicates that the oxidation consumed a large number of ATP enzymes during storage in NJ (*japonica* rice). The down-regulation of ubiquinone and other terpenoid quinone biosynthesis pathways in JZ (*indica* rice) is also related to the transfer of H^+^ during respiration. However, the degree of down-regulation does not significantly reduce the levels of the related proteins in the oxidative phosphorylation pathway. Li et al. [40] reported that the oxidative phosphorylation was enhanced during storage in *Coregonus peled*. Glyceraldehyde 3-phosphate dehydrogenase, malate dehydrogenase, and creatine kinase were three proteinases related to protein oxidative damage that showed a strongly significant correlation with deterioration in quality.

Proteins related to fatty acid metabolism include 3-oxoacyl-[acyl-carrier-protein] synthase, enoyl-[acyl-carrier-protein] reductase [NADH]2, acyl-[acyl-carrier-protein] hydrolase, methylcrotonoyl-CoA carboxylase subunit alpha, and two acyl carrier proteins (A3C125 and Q6ZJI9) (Table 3, No.8-12). Acetyl coenzyme A (CoA) is the precursor of fatty acid synthesis, and malonyl coenzyme catalyzed by acyl carrier protein is an essential substrate for fatty acid metabolism. Acyl-[acyl-carrier-protein] hydrolase releases fatty acids from the final products of fatty acid biosynthesis. Thus, the large consumption of acyl carrier protein, and the up-regulation of acyl-[acyl-carrier-protein] hydrolase suggested that fatty acids are continuously produced in NJ (Table 3, No.10). Lipidomic and metabolomic analyses revealed that the fatty acid metabolism pathway was the most active one in *japonica* rice after storage [14,15,16]. This result also supports our previous finding according to which *indica* rice was more stable under environmental changes than *japonica* rice [14].

High levels of starch and sucrose metabolism in stored rice were found in two varieties. The content of glycosyltransferase was increased in both varieties (Table 3, No.28 and 37), indicating that glucose was continuously consumed during storage. Another up-regulated DEP in the starch and sucrose metabolism of JZ was UTP-glucose-1-phosphate uridylyltransferase (Table 3, No.36). α-1,4 Glucan phosphorylase was down-regulated in NJ (Table 3, No. 38), while BGLU18, SUT2, and GBSSI were up-regulated (Table 3, No. 30,33 and 34). This may explain the higher amylose content in stored rice compared to fresh rice [41,42]. Nonetheless, PFK ALDO, phosphopyruvate hydratase, pyruvate kinase, and aldose 1-epimerase (Table 3, No. 27, 29, 31, 32, and 35) are only related to glycolysis/gluconeogenesis in NJ (*japonica* rice), and all of them were up-expressed in NJS. These results corroborate our previous study findings according to which storage increases the sugar metabolism of *japonica* rice, and accelerates the accumulation of organic acid [14]. Der Agopian et al. [43] compared the enzyme activity and protein levels related to starch conversion to sucrose in bananas stored at different temperatures. The results displayed that under any condition, the metabolism of starch and sucrose in bananas were significantly induced. A recent study investigating the quality of post-harvest kiwi fruit also showed that starch degradation and soluble sugar accumulation contributed to post-harvest flavor (ethanol) deterioration [44]. Therefore, the DEPs provide a foundation for screening potential biomarkers of quality deterioration in stored rice.

Glutelin, as a storage protein constituting more than 80% of rice, was generally associated with the quality of rice [45]. The glutelin family consists of four subfamilies of types A, B, C, and D. Type B includes five members of GluB-1/2/3/45. Adverse environment stress can accelerate the expression of glutelin type B proteins in the later stage of rice filling [46]. Zhao et al. [47] reported that the levels of glutelin type B proteins were increased in stored rice. Similarly, in this study, the expression of GluB-5 and GluB-5-like were significantly up-regulated in NJS and JZS, respectively (Table 3, No. 39 and 40).

Moreover, post-harvest operations before storage also greatly affect the quality of rice. Different forms of drying and processing play a critical role. An important goal of harvesting is to maintain the weight, avoid mechanical damage and changes in chemical composition, and prevent microbial and pest contamination [48]. The grain size of rice is decreased by the increase in external compression during drying. With the increased heating time, the internal pressure of rice increases and the central layer of grain expands. In this process, the mechanical stress of the grains is very high. When the grain surface cannot sustain its plasticity or elasticity, the grains crack or even fracture [49]. Regardless of the processing of incomplete rice grains (rice flour, rice noodles, etc.), shelling has a great impact on the storage of rice under room temperature [50]. Therefore, this study adopted the same post-processing method before storage to ensure the comparability of the processing groups.

**Figure 5:**
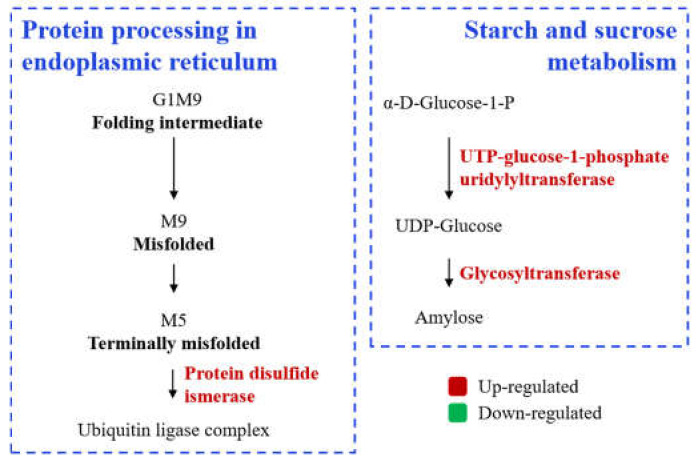
Storage drives alterations of metabolic pathways in Jianzhen 2 (indica rice).

**Figure 6:**
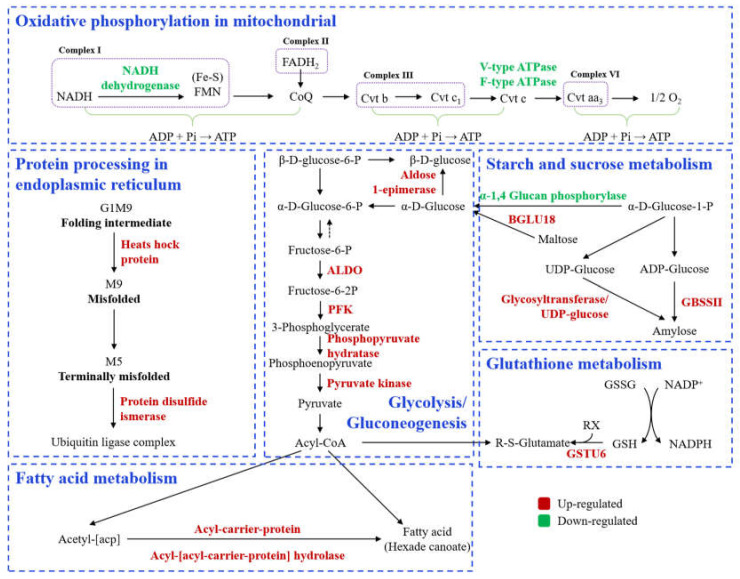
Storage drives alterations of metabolic pathways in Nanjing 9108 (*japonica* rice).

Compared to JZ (*indica* rice), the influence of storage on protein structure and profile in NJ (*japonica* rice) was more significant. Storage promoted protein synthesis and carbohydrate metabolism in JZ (*indica* rice) (Figure 5). However, storage can exacerbate redox homeostasis and oxidative phosphorylation, as a response to oxidative stress, glycolysis, fatty acid metabolism, starch and sucrose metabolism, and suppress the glutathione oxidation in NJ (*japonica* rice), resulting in lipid oxidation, abnormal gelatinization, and quality deterioration (Figure 6). The DEPs listed in Table 3 also provided a reference for the screening of potential biomarkers of quality deterioration in NJ and JZ rice during storage.

## 4. Conclusions

The impact of storage on the protein structure of NJ (*japonica* rice) was greater than on JZ (*indica* rice). Compared to freshly harvested rice, the levels of free sulfhydryl groups were significantly reduced in stored rice protein, and the content of disulfide bonds and surface hydrophobicity significantly improved. FTIR suggested that storage reduced the α-helix structure of the protein in both varieties. The changes in residues of the three secondary structures of the protein differed in JZ and NJ. Based on the proteomics analysis of TMT-labeled, 60 and 831 DEPs were identified in JZ and NJ, respectively. Storage-driven protein synthesis and carbohydrate metabolism was found in both varieties. They were specifically associated with redox homeostasis, oxidative balance, glycolysis, fatty acid metabolism, starch and sucrose metabolism, and glutathione metabolism in NJ (*japonica* rice). The results provide an insight into the mechanism of rice quality and its deterioration during storage. However, this study only investigated a single variety of *japonica* rice and a single variety of *indica* rice. Additional studies involving different varieties are needed to establish the alterations of proteomic profiles, as well as to identify the relevant biomarkers during rice storage.

## Figures and Tables

**Table 1:** Influence of storage on secondary structural contents of rice protein in two varieties (n = 3, α = 0.05).

Sample	α-Helix/%	β-Sheet/%	β-Turn/%	Random Coil/%
NJF	20.6 ± 0.2	37.3 ± 0.2	11.2 ± 0.2	30.8 ± 0.3
NJS	17.8 ± 0.2 *	21.6 ± 0.5 *	13.1 ± 0.2 *	47.5 ± 0.4 *
JZF	32.4 ± 0.7	17.1 ± 0.5	12.9 ± 0.7	37.6 ± 0.5
JZS	30.7 ± 0.5 *	19.9 ± 0.8 *	11.6 ± 0.1 *	37.8 ± 0.3

*: means the stored group is significantly different compared with the fresh group in the same variety.

**Table 2:** Common DEPs of two varieties.

No.	Protein ID	NJS vs. NJF			JZS vs. JZF			KEGG Pathway Name
		FC	*p*-Value	Regulated	FC	*p*-Value	Regulated	
1	Q67VS7	2.013	0.004	up	1.313	0.046	up	
2	C7J9I2	1.653	0.001	up	1.518	0.028	up	/
3	Q0J8Q8	1.502	0.000	up	1.322	0.005	up	/
4	Q6AUV3	1.467	0.008	up	1.249	0.005	up	/
5	Q6ATY6	1.409	0.011	up	1.248	0.025	up	Purine metabolism Pyrimidine metabolism Metabolic pathways RNA polymerase
6	B9EZ14	1.387	0.003	up	1.266	0.014	up	/
7	Q7XQF0	1.376	0.007	up	1.252	0.018	up	Ubiquitin-mediated proteolysis
8	B9F2Z5	1.321	0.005	up	1.212	0.034	up	/
9	B9G0M2	1.282	0.001	up	1.212	0.025	up	Ubiquitin-mediated proteolysis
10	Q6ZJ18	1.248	0.001	up	1.233	0.031	up	/
11	Q2QPW3	0.809	0.001	down	0.802	0.025	down	/
12	B9G505	0.649	0.001	down	0.771	0.044	down	Protein export
13	B9ESY3	0.776	0.000	down	1.435	0.046	up	Spliceosome
14	A0A0P0Y2R7	2.199	0.000	up	0.667	0.012	down	
15	Q8S7K1	1.302	0.000	up	0.791	0.013	down	/

**Table 3:** Partial DEPs related to quality deterioration in two varieties during storage.

No.	Protein ID	Description	Gene	NJS vs. NJF			JZS vs. JZF		
				FC	*p*-Value	Regulated	FC	*p*-Value	Regulated
Redox Homeostasis/Stress-Related									
1	Q655N4	Putative 70 kDa heat-shock protein	OsJ_22372	1.344	0.006	up	/	/	/
2	Q6K7E9	18.6 kDa class III heat shock protein	HSP18.6	1.322	0.006	up	1.110	0.102	/
3	Q6AUW3	22.3 kDa class VI heat shock protein	HSP22.3	1.228	0.030	up	1.177	0.334	/
4	Q943E6	16.9 kDa class I heat shock protein 2	HSP16.9B	1.221	0.047	up	0.856	0.292	/
5	Q7XRB5	Protein disulfide isomerase-like 1-2	PDIL1-2	2.197	0.026	up	1.038	0.787	/
6	K4FHN8	Protein disulfide-isomerase	Unknown	1.030	0.604	/	1.732	0.001	up
7	Q945W9	Glutathione S-transferase GSTU6	LOC_Os10g38590	1.396	0.003	up	1.037	0.401	/
Oxidative phosphorylation in mitochondria									
8	Q0JKB4	F-type H^+^-transporting ATPase subunit β	Os01g0685800	0.659	0.019	down	0.856	0.018	/
9	Q8GTK7	F-type H^+^-transporting ATPase subunit δ	Os07g0495200	0.673	0.000	down	0.972	0.735	/
10	Q7XXS0	F-type H^+^-transporting ATPase subunit d	Os08g0478200	0.759	0.010	down	1.062	0.585	/
11	Q8RU25	F-type H^+^-transporting ATPase subunit γ	OSJNAa0053D03.3	0.831	0.045	down	0.874	0.083	/
12	B9F0H4	V-type proton ATPase proteolipid subunit	Os02g0550100	0.798	0.020	down	/	/	/
13	Q2F948	NADH-ubiquinone oxidoreductase chain 5	nad5	0.668	0.007	down	/	/	/
14	Q8HCQ0	NADH-ubiquinone oxidoreductase chain 3	nad3	0.780	0.039	down	1.158	0.300	/
15	Q8HCM1	NADH dehydrogenase subunit 2	nad2	0.807	0.019	down	1.052	0.524	/
16	Q6K9W2	NADH dehydrogenase [ubiquinone] 1 α subcomplex subunit 9	Os02g0816800	0.667	0.002	down	0.992	0.729	/
17	A0A5S6RA53	NADH dehydrogenase [ubiquinone] 1 α subcomplex subunit 12	Os10g0579300	0.725	0.000	down	0.974	0.643	/
18	Q8GS72	NADH dehydrogenase [ubiquinone] 1 α subcomplex subunit 6	Os07g0640100	0.774	0.002	down	1.028	0.692	/
19	A0A5S6RAL2	NADH dehydrogenase [ubiquinone] 1 α subcomplex subunit 6	Os04g0310500	0.783	0.002	down	1.004	0.939	/
20	Q0J7V0	NADH dehydrogenase [ubiquinone] iron-sulfur protein 6	P0577B11.122	0.748	0.006	down	0.982	0.658	/
21	Q6ZJ19	NADH dehydrogenase [ubiquinone] iron-sulfur protein 5-B	Os08g0556600	0.756	0.014	down	1.126	0.086	/
Fatty acid metabolism									
22	B7F6I0	3-oxoacyl-[acyl-carrier-protein] synthase	Os04g0445700	1.563	0.002	up	1.078	0.161	/
23	Q6H5J0	Enoyl-[acyl-carrier-protein] reductase [NADH] 2	Os09g0277800	1.492	0.000	up	1.012	0.680	/
24	A3CDM0	Acyl-[acyl-carrier-protein] hydrolase	Os11g0659500	1.247	0.010	up	1.041	0.147	/
25	A3C125	Acyl carrier protein	OsJ_30173	0.770	0.022	down	1.096	0.300	/
26	Q6ZJI9	Acyl carrier protein	Os08g0549300	0.642	0.010	down	1.064	0.541	/
Energy/carbohydrate metabolism									
27	A0A0P0UZU8	ATP-dependent 6-phosphofructokinase	PFK	1.289	0.009	up	1.063	0.088	/
28	A0A0P0W2E0	Glycosyltransferase	Os03g0693600	1.768	0.000	up	1.135	0.178	/
29	Q40677	Fructose-bisphosphate aldolase	ALDO	1.495	0.000	up	0.934	0.086	/
30	Q7XSK0	β-glucosidase 18	BGLU18	1.457	0.001	up	0.934	0.305	/
31	B7FA07	Phosphopyruvate hydratase	Os09g0375000	1.340	0.001	up	0.954	0.387	/
32	Q8S7N6	Pyruvate kinase	LOC_Os10g42100	1.332	0.017	up	0.925	0.717	/
33	Q0ILJ3	Sucrose transport protein SUT2	SUT2	1.300	0.009	up	1.032	0.402	/
34	Q8GTK0	Starch synthase	GBSSII	1.297	0.021	up	/	/	/
35	A0A0P0VKS3	Aldose 1-epimerase	Os02g0575800	1.200	0.003	up	0.972	0.589	/
36	Q0E4I5	UTP-glucose-1-phosphate uridylyltransferase	Os02g0117700	/	/	/	1.378	0.020	up
37	Q6ESW3/ A3A4Z8	Glycosyltransferase	Os02g0242900	/	/	/	1.412	0.000	up
38	Q10CK4	α-1,4 glucan phosphorylase	LOC_Os03g55090	0.470	0.001	down	0.812	0.458	/
Gluetin									
39	Q84X94	Glutelin	GluB-5	1.473	0.011	up	0.971	0.863	/
40	A0A0N7KF02/ Q6ESW6	Glutelin	GluB-5-like, Os02g0242600	1.076	0.378	/	1.432	0.028	up

## Data Availability

Data is contained within the article or supplementary material.

## Supplementary Materials

The following supporting information can be downloaded at: https://www.mdpi.com/article/10.3390/foods11213541/s1, Figure S1: Effect of storage on Fourier transform infrared spectroscopy of rice protein. (a) NJF; (b) NJS; (c) JZF; (d) JZS; Figure S2: The chromatograms of LC-MS of 10 peptide fractions of rice sample; Figure S3: Tolerance distribution of parent ion mass in JZ (a) and NJ (b); Figure S4: Distribution map of peptide length range in JZ (a) and NJ (b); Figure S5: Protein coverage distribution in JZ (a) and NJ (b); Figure S6: Protein variation between stored rice compared with fresh rice. (a) Up-regulation/down-regulation of the top 10 DEPs in JZ; (b) Up-regulation/down-regulation of the top 10 DEPs in NJ; Table S1: Liquid chromatography gradient elution program of separation of polypeptide fraction; Table S2: Liquid chromatography gradient elution program; Table S3: Parameters of Proteome Discoverer software; Table S4a: Complete list of the measured proteins in Jianzhen 2; Table S4b: Complete list of the measured proteins in Nanjing 9108; Table S5a: Detailed information of the DEPs in Jianzhen 2; Table S5b: Detailed information of the DEPs in Nanjing 9108.

## References

[1] Zhou Z., Robards K., Helliwell S., Blanchard C. (2002). Ageing of Stored Rice: Changes in Chemical and Physical Attributes. J. Cereal Sci..

[2] Saikrishna A., Dutta S., Subramanian V., Moses J.A., Anandharamakrishnan C. (2018). Ageing of rice: A review. J. Cereal Sci..

[3] Xu J., Liu K., Zhang C. (2021). Electronic nose for volatile organic compounds analysis in rice aging. Trends Food Sci. Technol..

[4] Chen Z., Li P., Du Y., Jiang Y., Cai M., Cao C. (2021). Dry cultivation and cultivar affect starch synthesis and traits to define rice grain quality in various panicle parts. Carbohydr. Polym..

[5] Pan T., Zhao L., Lin L., Wang J., Liu Q., Wei C. (2017). Changes in kernel morphology and starch properties of high-amylose brown rice during the cooking process. Food Hydrocoll..

[6] Ong M.H., Blanshard J.M.V. (1995). Texture determinants of cooked, parboiled rice. II: Physicochemical properties and leaching behaviour of rice. J. Cereal Sci..

[7] Zhao Q., Lin J., Wang C., Yousaf L., Xue Y., Shen Q. (2021). Protein structural properties and proteomic analysis of rice during storage at different temperatures. Food Chem..

[8] Shi L., Zhang X., Sun H., Cao X., Liu J., Zhang Z. (2019). Relationship of grain protein content with cooking and eating quality as affected by nitrogen fertilizer at late growth stage for different types of rice varieties. Chin. J. Rice Sci..

[9] Chen Y., Jiang W., Jiang Z., Chen X., Cao J., Dong W., Dai B. (2015). Changes in Physicochemical, Structural, and Sensory Properties of Irradiated Brown Japonica Rice during Storage. J. Agric. Food Chem..

[10] Guo Y., Cai W., Tu K., Tu S., Wang S., Zhu X., Zhang W. (2012). Infrared and Raman Spectroscopic Characterization of Structural Changes in Albumin, Globulin, Glutelin, and Prolamin during Rice Aging. J. Agric. Food Chem..

[11] Itze-Mayrhofer C., Brem G. (2020). Quantitative proteomic strategies to study reproduction in farm animals: Female reproductive fluids. J. Proteom..

[12] Liu Y., Liu J., Wang A., Wang R., Sun H., Strappe P., Zhou Z. (2020). Physiological and proteomic analyses provide insights into the rice yellowing. J. Cereal Sci..

[13] Xiao R., Li L., Ma Y. (2019). A label-free proteomic approach differentiates between conventional and organic rice. J. Food Compos. Anal..

[14] Wang Q., Zhang D., Zhao L., Liu J., Shang B., Yang W., Duan X., Sun H. (2022). Metabolomic Analysis Reveals Insights into Deterioration of Rice Quality during Storage. Foods.

[15] Zhang D., Duan X., Shang B., Hong Y., Sun H. (2021). Analysis of lipidomics profile of rice and changes during storage by UPLC-Q-extractive orbitrap mass spectrometry. Food Res. Int..

[16] Zhang D., Zhao L., Wang W., Wang Q., Liu J., Wang Y., Liu H., Shang B., Duan X., Sun H. (2022). Lipidomics reveals the changes in non-starch and starch lipids of rice (*Oryza sativa* L.) during storage. J. Food Compos. Anal..

[17] Wu X., Li F., Wu W. (2020). Effects of oxidative modification by 13-hydroperoxyoctadecadienoic acid on the structure and functional properties of rice protein. Food Res. Int..

[18] Kachuk C., Stephen K., Doucette A. (2015). Comparison of sodium dodecyl sulfate depletion techniques for proteome analysis by mass spectrometry. J. Chromatogr. A.

[19] Wiśniewski J.R., Zougman A., Nagaraj N., Mann M. (2009). Universal sample preparation method for proteome analysis. Nat. Methods.

[20] Schöneich C. (2016). Thiyl radicals and induction of protein degradation. Free Radic. Res..

[21] Sun W., Zhou F., Sun D.-W., Zhao M. (2013). Effect of Oxidation on the Emulsifying Properties of Myofibrillar Proteins. Food Bioprocess Technol..

[22] Wang C., Wang J., Zhu D., Hu S., Kang Z., Ma H. (2020). Effect of dynamic ultra-high pressure homogenization on the structure and functional properties of whey protein. J. Food Sci. Technol..

[23] Azizi R., Capuano E., Nasirpour A., Pellegrini N., Golmakani M.-T., Hosseini S.M.H., Farahnaky A. (2019). Varietal differences in the effect of rice ageing on starch digestion. Food Hydrocoll..

[24] Hamaker B.R., Griffin V.K. (1993). Effect of disulfide bond-containing protein on rice starch gelatinization and pasting. Cereal Chem..

[25] Ren J., Li S. (2016). Effects of thermal treatment on structure and surface hydrophobicity of sunflower seed protein isolate. China Oils Fats.

[26] Zhou X., Liu Y., Li X., Yu J. (2010). Effects of protein-glutaminase on the molecular structure and functional properties of rice glutelin. J. Chin. Inst. Food Sci. Technol..

[27] Zhang B., Li H., Li F., Zhou Q., Wu X., Wu W. (2022). Effects of rice bran phenolics on the structure of rice bran protein under different degrees of rancidity. LWT.

[28] Hou F., Ding W., Qu W., Oladejo A.O., Xiong F., Zhang W., He R., Ma H. (2017). Alkali solution extraction of rice residue protein isolates: Influence of alkali concentration on protein functional, structural properties and lysinoalanine formation. Food Chem..

[29] Singh T.P., Sogi D.S. (2018). Comparative study of structural and functional characterization of bran protein concentrates from superfine, fine and coarse rice cultivars. Int. J. Biol. Macromol..

[30] Gao J., Fu H., Zhou X., Chen Z., Luo Y., Cui B., Chen G., Liu J. (2016). Comparative proteomic analysis of seed embryo proteins associated with seed storability in rice (*Oryza sativa* L) during natural aging. Plant Physiol. Biochem..

[31] Hu M. (2017). Study on the Quality Deterioration Mechanism of Tibetan Hulless Barley under Different Storage Conditions.

[32] Onda Y., Kobori Y. (2014). Differential activity of rice protein disulfide isomerase family members for disulfide bond formation and reduction. FEBS Open Bio.

[33] Cao Z. (2014). Effects of High Temperature on Rice (Oryza sativa L.) Floral Injury and Grain Quality in Relation to Carbon and Nitrogen Metabolism.

[34] Houston N.L., Fan C., Xiang Q.-Y., Schulze J.-M., Jung R., Boston R.S. (2005). Phylogenetic Analyses Identify 10 Classes of the Protein Disulfide Isomerase Family in Plants, Including Single-Domain Protein Disulfide Isomerase-Related Proteins. Plant Physiol..

[35] Xu M., He D., Teng H., Chen L., Song H., Huang Q. (2018). Physiological and proteomic analyses of coix seed aging during storage. Food Chem..

[36] Cao Q., Lv W., Jiang H., Chen X., Wang X., Wang Y. (2022). Genome-wide identification of glutathione S-transferase gene family members in tea plant (*Camellia sinensis*) and their response to environmental stress. Int. J. Biol. Macromol..

[37] Bhattacharya K.R., Bhattacharya K.R. (2013). 5—Ageing of Rice. Rice Quality: A Guide to Rice Properties and Analysis.

[38] Liu J., Hu Z., Liu D., Zheng A., Ma Q. (2023). Glutathione metabolism-mediated ferroptosis reduces water-holding capacity in beef during cold storage. Food Chem..

[39] Ajadi A.A., Cisse A., Ahmad S., Yifeng W., Yazhou S., Shufan L., Xixi L., Bello B.K., Tajo S.M., Xiaohong T. (2020). Protein Phosphorylation and Phosphoproteome: An Overview of Rice. Rice Sci..

[40] Li X., Deng X., Guo X., Wei Y., Zhao Y., Guo X., Zhu X., Zhang J., Hu L. (2022). Two-dimensional gel analysis to investigate the effect of hydroxyl radical oxidation on freshness indicator protein of Coregonus peled during 4 °C storage. LWT.

[41] Huang Y.-C., Lai H.-M. (2014). Characteristics of the starch fine structure and pasting properties of waxy rice during storage. Food Chem..

[42] Wu P., Li C., Bai Y., Yu S., Zhang X. (2019). A starch molecular basis for aging-induced changes in pasting and textural properties of waxy rice. Food Chem..

[43] Der Agopian R.G., Peroni-Okita F.H.G., Soares C.A., Mainardi J.A., do Nascimento J.R.O., Cordenunsi B.R., Lajolo F.M., Purgatto E. (2011). Low temperature induced changes in activity and protein levels of the enzymes associated to conversion of starch to sucrose in banana fruit. Postharvest Biol. Technol..

[44] Huan C., Du X., Wang L., Kebbeh M., Li H., Yang X., Shen S., Zheng X. (2021). Transcriptome analysis reveals the metabolisms of starch degradation and ethanol fermentation involved in alcoholic off-flavour development in kiwifruit during ambient storage. Postharvest Biol. Technol..

[45] Amagliani L., O’Regan J., Kelly A.L., O’Mahony J.A. (2017). The composition, extraction, functionality and applications of rice proteins: A review. Trends Food Sci. Technol..

[46] Tang S., Chen W., Liu W., Zhou Q., Zhang H., Wang S., Ding Y. (2018). Open-field warming regulates the morphological structure, protein synthesis of grain and affects the appearance quality of rice. J. Cereal Sci..

[47] Zhao Q., Xue Y., Shen Q. (2020). Changes in the major aroma-active compounds and taste components of Jasmine rice during storage. Food Res. Int..

[48] Paulo C.C., Carlos H.P.F., Jean C.H. (2016). Adjustment of mathematical models and quality of soybean grains in the drying with high temperatures. Rev. Bras. Eng. Agrícola Ambient..

[49] Müller A., Nunes M.T., Maldaner V., Coradi P.C., de Moraes R.S., Martens S., Leal A.F., Pereira V.F., Marin C.K. (2022). Rice Drying, Storage and Processing: Effects of Post-Harvest Operations on Grain Quality. Rice Sci..

[50] Qiu S., Kawamura S., Fujikawa S., Doi T. (2014). Long-term storability of rough rice and brown rice under different storage conditions. Eng. Agric. Environ. Food.

