# Supplementary material for: Storage Drives Alterations of Proteomic and Protein Structural Properties in Rice (Oryza sativa L.)
Source: Foods. 2022 Nov 7;11(21):3541. doi: 10.3390/foods11213541 (PMC9658062; doi:10.3390/foods11213541)
Supplement: Supplementary file 1 [file foods-11-03541-s001.zip › Supplementary material-01.pdf]

Storage drives alterations of proteomic and protein structural properties in rice (*Oryza sativa L.*)

Qian Wang, Dong Zhang, Jianlei Liu, Bo Shang, Xiaolaing Duan, Hui Sun*

Academy of National Food and Strategic Reserves Administration, Beijing 100037, PR China

*Corresponding author:

Dr. Hui Sun

Academy of National Food and Strategic Reserves Administration

No. 11, Baiwanzhuang street, Xicheng district, Beijing, P. R. China

Ph: 86-13601375755 E-mail: sh@ags.ac.cn

Supplementary Materials

Table S1 Liquid chromatography gradient elution program of separation of polypeptide fraction.

RT (min)	Flow Rate (mL/min)	Mobile Phase A (%)	Mobile Phase B (%)
0	1	97	3
10	1	95	5
30	1	80	20
48	1	60	40
50	1	50	80
53	1	30	70
54	1	0	100

Table S2 Liquid chromatography gradient elution program.

RT (min)	Flow Rate (nL/min)	Mobile Phase A (%)	Mobile Phase B (%)
0	600	94	6
2	600	85	15
48	600	60	40
50	600	50	50
51	600	45	55
60	600	0	100

Table S3 Parameters of Proteome Discoverer software.

Item	Value
Type of Quantification	Reporter Quantification(TMT)
Enzyme	Trypsin
Max.Missed Cleavage Sites	2
Precursor Mass Tolerance	10 ppm
Fragment Mass Tolerance	0.02 Da
Dynamic Modification	Oxidation/+15.995 Da (M) and TMT /+229.163 Da (K,Y)
N-Terminal Modification	Acetyl/+42.011 Da (N-Terminal) and TMT /+229.163 Da (N-Terminal)
Static Modification	Carbamidomethyl/+57.021 Da (C)

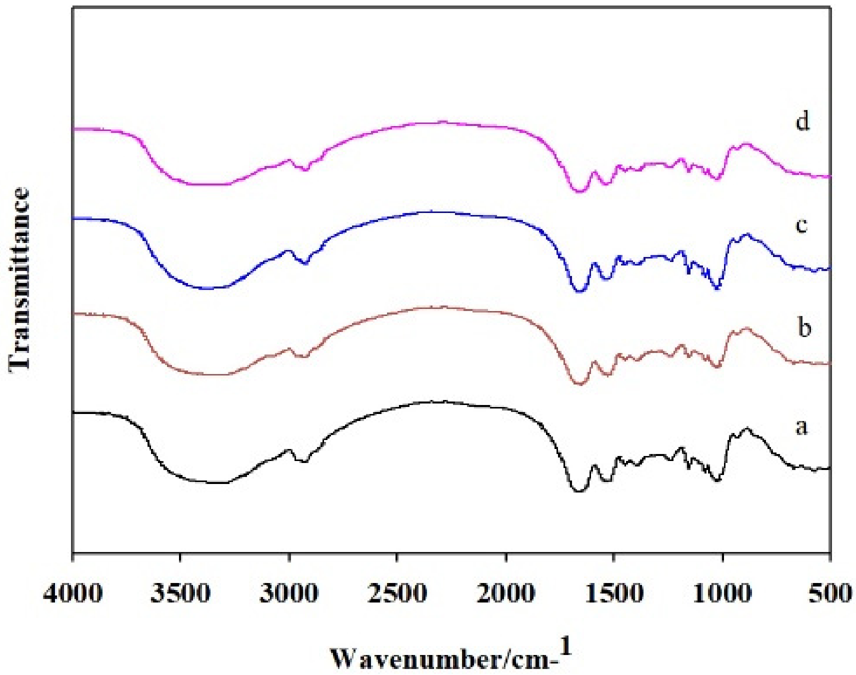

Figure S1. Effect of storage on Fourier transform infrared spectroscopy of rice protein. (a) NJF; (b) NJS; (c) JZF; (d) JZS.

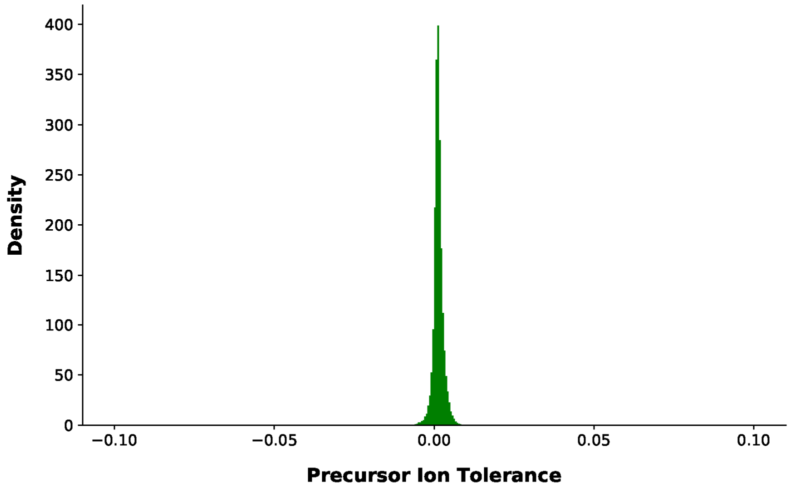

(a)

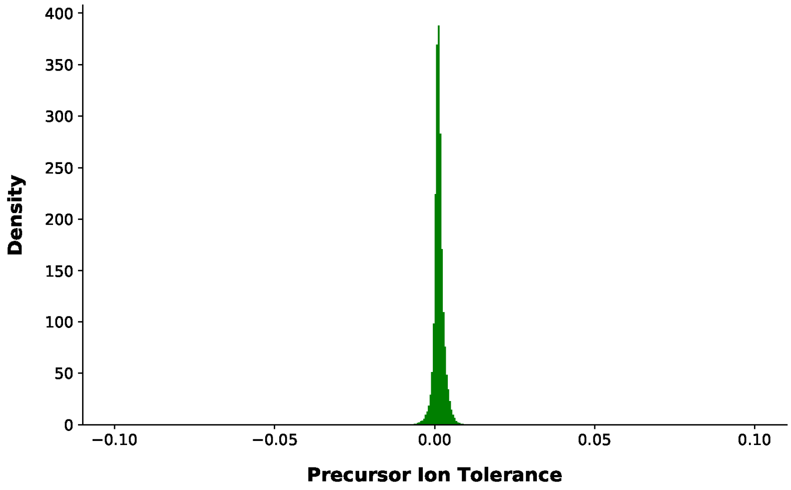

(b)

Figure S3. Tolerance distribution of parent ion mass in JZ (a) and NJ (b).

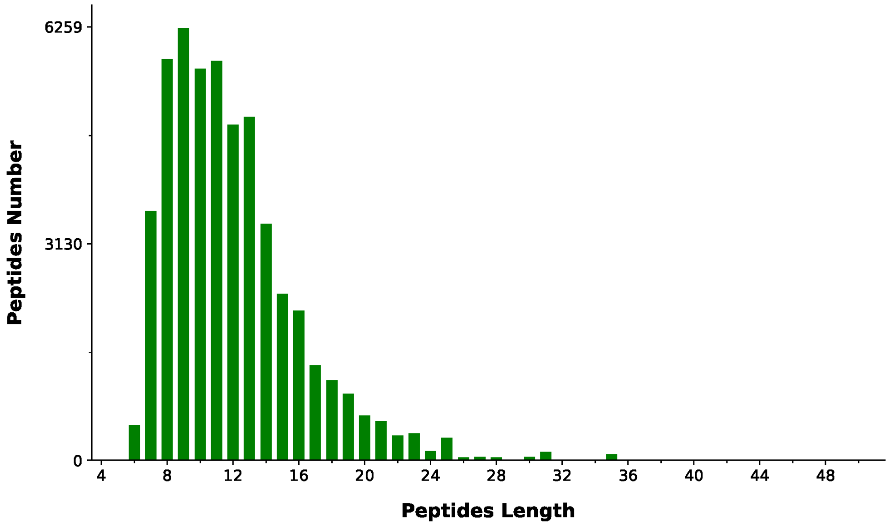

(a)

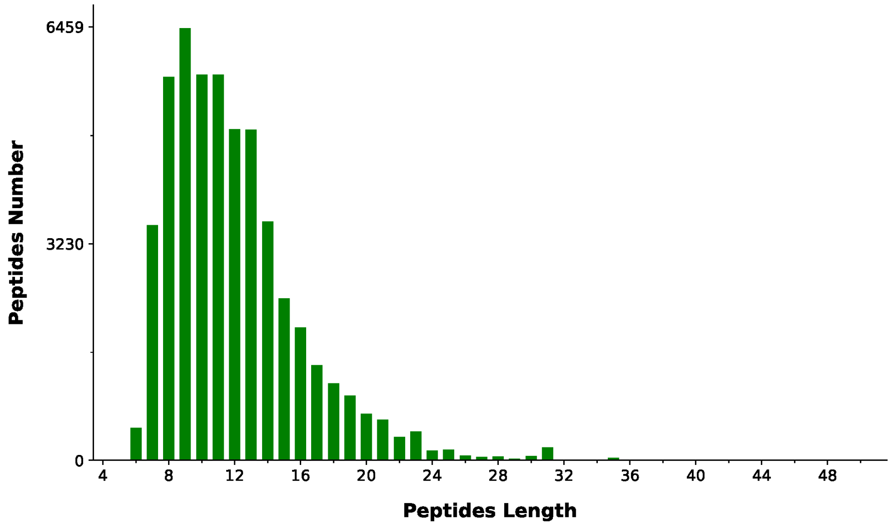

(b)

Figure S4. Distribution map of peptide length range in JZ (a) and NJ (b).

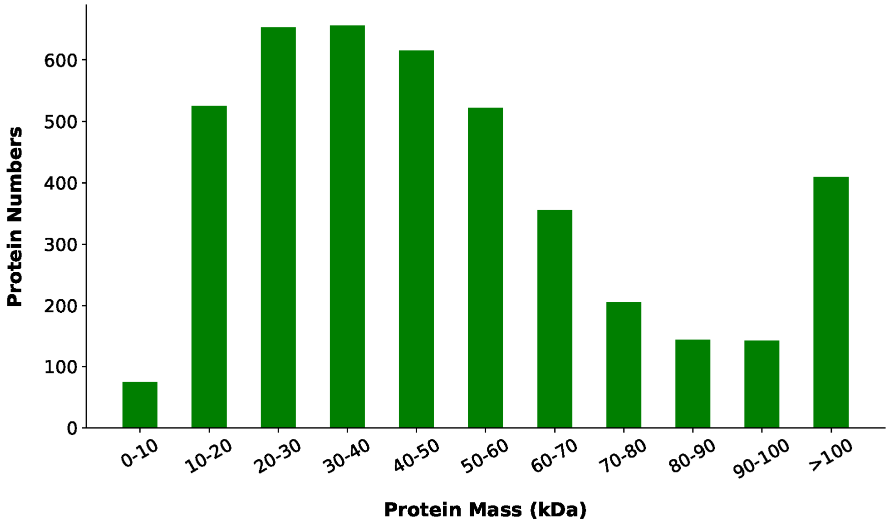

(a)

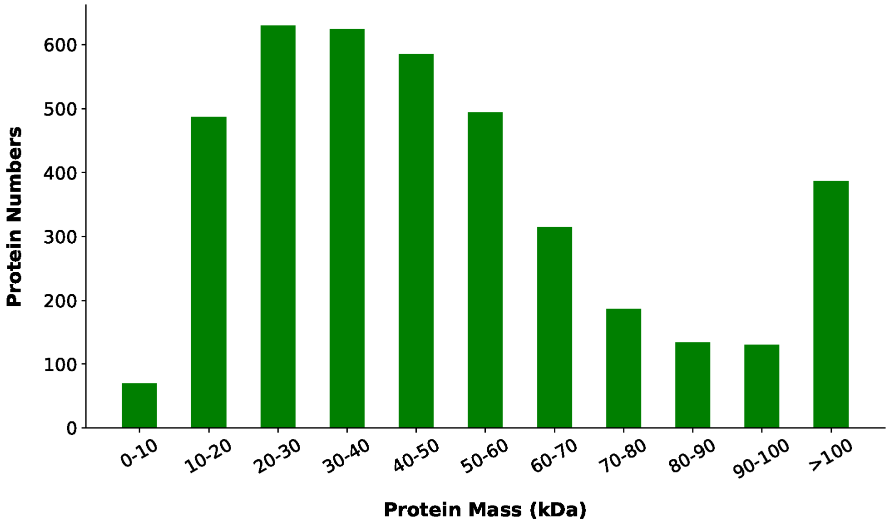

(b)

Figure S5. Protein coverage distribution in JZ (a) and NJ (b).

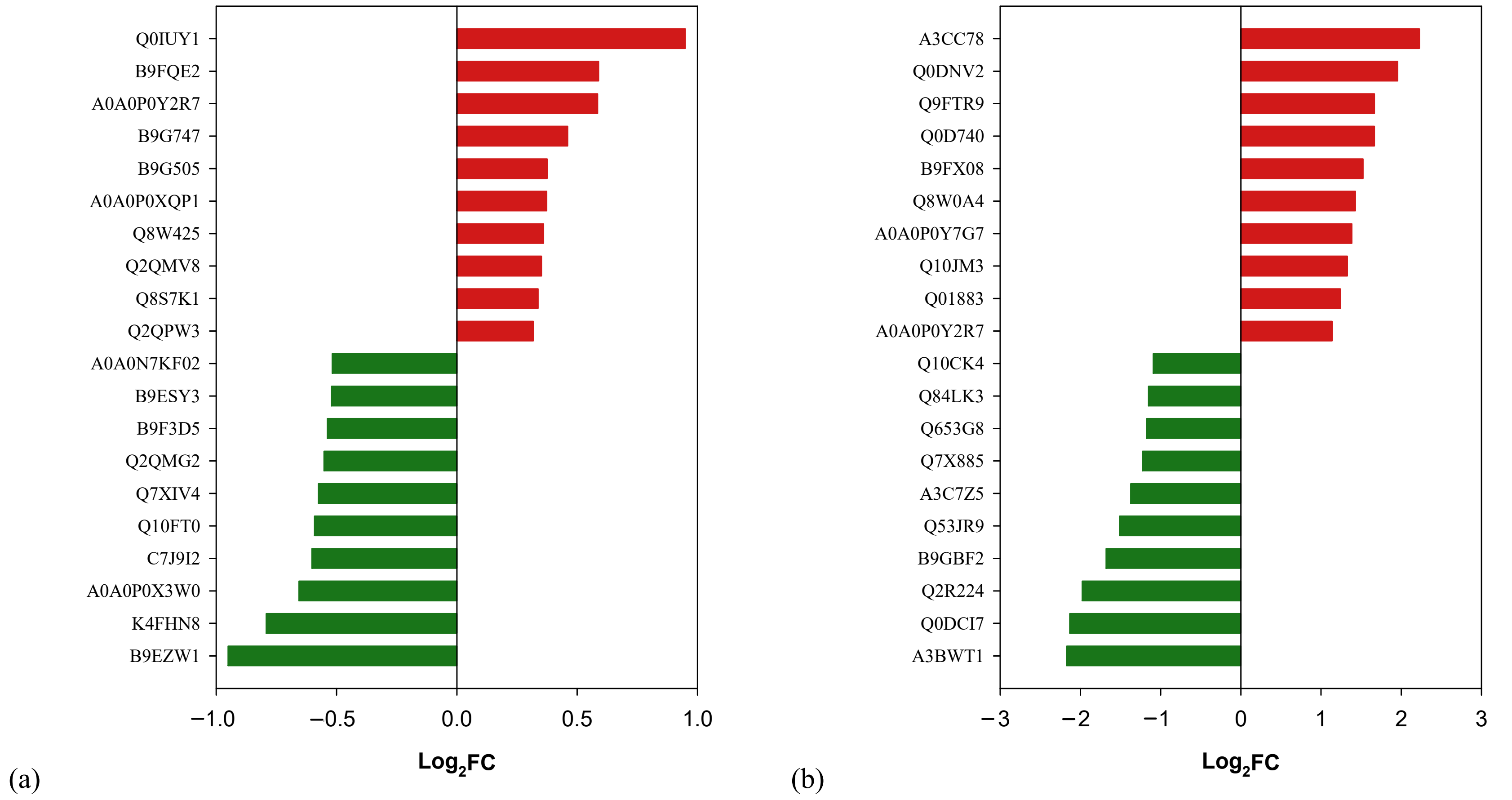

Figure S6. Protein variation between stored rice compared with fresh rice. (a) Up-regulation/down-regulation of the top 10 DEPs in JZ; (b) Up-regulation/down-regulation of the top 10 DEPs in NJ.

The mass spectrometry proteomics data have been deposited to the ProteomeXchange Consortium via the PRIDE [1] partner repository with the dataset identifier PXD037810.

[1] http://www.ebi.ac.uk/pride
